# Coronavirus disease 2019 and peripheral blood eosinophil counts: a retrospective study

**DOI:** 10.1007/s15010-021-01710-w

**Published:** 2021-10-08

**Authors:** Marjella Eijmael, Nicky Janssens, Saskia le Cessie, Yordi van Dooren, Ted Koster, Faiz Karim

**Affiliations:** 1 Department of Internal Medicine, Groene Hart Ziekenhuis, Bleulandweg 10, 2803 HH Gouda, The Netherlands; 2 Department of Clinical Epidemiology and Biomedical Data Sciences, Leiden University, Leiden, The Netherlands; 3 Department of Pulmonary Medicine, Groene Hart Ziekenhuis, Gouda, The Netherlands

**Keywords:** Eosinopenia, Peripheral blood eosinophil count, COVID-19 disease, SARS-CoV2, Complete blood count, Lymphopenia

## Abstract

**Purpose:**

Eosinopenia has been described in COVID-19. With this study, we aim to study the peripheral blood eosinophil counts in COVID-19 patients and to investigate whether there is an association between the peripheral blood eosinophil counts and disease severity of COVID-19.

**Methods:**

We revised the electronical medical records of confirmed COVID-19 patients with polymerase chain reaction (PCR) assays in the Groene Hart Ziekenhuis, Gouda, The Netherlands. We divided patients in mild, moderate and severe groups based on clinical severity of COVID-19. Clinical severity was based on the therapy needed and the outcome of patients. We compared clinical characteristics, laboratory results and outcome between the three groups.

**Results:**

Of the 230 patients included in this study, the mild, moderate and severe groups consisted of 16.5%, 45.7% and 37.8% of the included patients, respectively. The mean age was 68 years (IQR 57–78). 63% of patients were male. A significant decrease in the peripheral eosinophil counts was found corresponding to the increase of COVID-19 severity. In the mild, moderate and severe groups, the percentage of patients with eosinopenia was 73.7%, 86.7% and 94.3%, respectively (*p* value 0.002).

**Conclusion:**

Eosinopenia is significantly more frequent present in patients with a severe COVID-19.

## Introduction

Coronavirus disease 2019 (COVID-19) is a current pandemic disease for over a year now. Eosinopenia has previously been described in COVID-19, however, their role in this disease remains under debate [1, 2]. With this study, we aimed to examine the peripheral blood eosinophil levels in patients and to investigate whether there is an association between eosinopenia and the disease severity of COVID-19.

## Methods

We conducted a single center, retrospective cohort study. Data were collected from all polymerase chain reaction (PCR) confirmed COVID-19 patients from the start of the pandemic, March 2020, until the 31th of October 2020, seen at the emergency department or hospital ward in the ‘Groene Hart Ziekenhuis’ (GHZ) in Gouda, the Netherlands. The study was approved by the local Medical Ethics Review Committee Scientific Committee (no. G20.179).

Complete blood count was collected at admission or at the first day of a positive test, if patients were tested later during their hospital admission. Measurements included the numbers of leukocytes, immune granulocytes, neutrophils, lymphocytes, monocytes, eosinophils, basophils and C-reactive protein (CRP). Lymphopenia was defined as lymphocyte counts below 0.8 × 10^9^ cells/L. Eosinopenia was defined as eosinophil counts below 0.04 × 10^9^ cells/L. We excluded patients younger than 18 year old, patients with a pre-existing pancytopenia and patients using systemic glucocorticosteroids [3]. Clinical characteristics, laboratory findings and different kind of (oxygen) therapies of all patients were retrieved from the electronic medical record system. Furthermore, disease days and duration of hospital admission were recorded. Re-admission was registered if patients were re-admitted to the GHZ, after being hospitalized in the GHZ or another hospital, due to COVID-19 after being previously discharged.

The patients were divided in three groups according to disease severity. Group 1, also referred to as ‘mild’, included asymptomatic patients and patients who did not require oxygen therapy. Group 2, also referred to as ‘moderate’, included patients who received some supplemental oxygen but less than 15L. Group 3, also referred to as ‘severe’, included patients who needed the maximum of 15L oxygen with a non-rebreathing mask, optiflow high-flow nasal oxygen, intubation, intensive care unit admission or who had a COVID-19-related death in the hospital or else after being sent to for example a caring home or hospice for palliative care and died within 14 days.

## Results

**Fig. 1:**
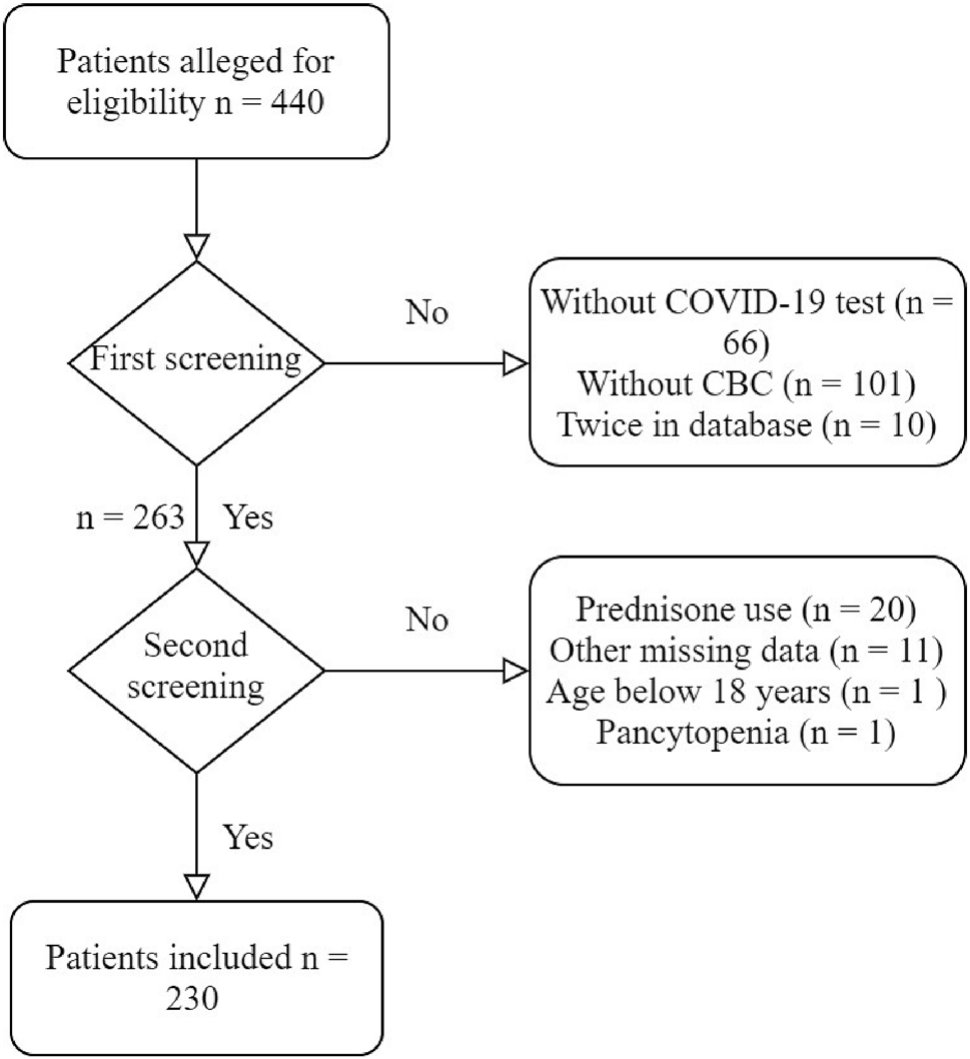
Patient inclusion and exclusion

**Fig. 2:**
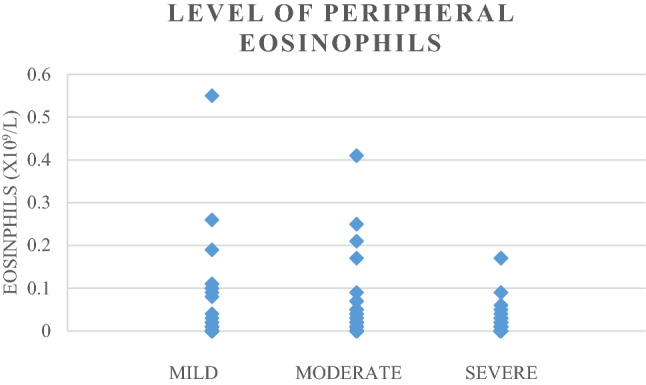
Diversion of the level of peripheral eosinophil counts in each COVID-19 severity group

A total of 440 patients with suspected COVID-19 were seen in our hospital. Patients with unconfirmed COVID-19 (*n* = 66) and patients who missed complete blood count (*n* = 101) were excluded. Patients were also excluded if they used prednisone, had an age below 18 years or had a known pancytopenia before COVID-19 infection or missed other relevant data. Eventually, a total 230 patients were included (Fig. 1). Table 1 shows the demographic and baseline characteristics in the different severity groups. A total of 38 (16.5%) mild, 105 (45.7%) moderate and 87 (37.8%) severe patients were included. Complete blood counts demonstrated that patients with a more severe disease course had significantly higher leukocyte and neutrophil counts in the peripheral blood (Table 2). There was no significant difference between the number of patients with lymphopenia between the groups, with 31.6% in the mild group, 29.5% in the moderate group and 37.9% in the severe group (Table 3). As expected the CRP level increased significantly with disease severity of COVID-19 (Table 2, *p* value < 0.001). A significant decrease in the level of eosinophil counts was observed between the groups (Fig. 2, *p* value 0.004). The median (IQR) level of eosinophils was 0.01 (0.00–0.05) × 10^9^ cells/L in the mild group, 0.00 (0.00–0.02) × 10^9^ cells/L in the moderate group and 0.00 (0.00–0.01) × 10^9^ cells/L in the severe group. Regression analysis, adjusting for age, sex and the presence of comorbidity, showed that the level of eosinophils still significantly differed between the severity groups (*p* value 0.028). The number of patients with eosinopenia differed significantly between the severity groups (Table 3). In the mild, moderate and severe groups, the percentage of patients with eosinopenia was, respectively, 73.7%, 86.7% and 94.3% (Table 3, *p* value 0.002). This demonstrates that the number of patients with eosinopenia rose with increasing severity of COVID-19. These numbers suggest a relation between eosinopenia and severity of COVID-19. Lymphopenia was present in only 33% of the patients, which is lower compared to previous studies where lymphopenia was found in 70–78% of the patients [1, 2, 4]. This can partially be explained by a higher cutoff point of lymphopenia (below 1.1 × 10^9^ cells/L), whereas we used a cut off of 0.8 × 10^9^ cells/L, according to our local guidelines.

**Table 1:** Demographic and baseline characteristics of COVID-19 PCR confirmed patients included in the study population, stratified by severity group

	Mild	Moderate	Severe	Total	*p* value
	(*n* = 38)	(*n* = 105)	(*n* = 87)	(*n* = 230)	
All					
Age y, range	18–88	21–96	30–91	18–96	
Age y, median (IQR)	68 (54–79)	65 (55–76)	71 (59–80)	68 (57–78)	0.03^a^
Women—*n* (%)	22 (58)	40 (38)	23 (26)	85 (37)	0.001
Age y, range	18–88	33–96	46–91	18–96	
Age y, median (IQR)	68 (52–80)	71 (56–79)	72 (63–83)	71 (56–80)	0.06^a^
Men—*n* (%)	16 (42)	65 (62)	64 (74)	145 (63)	0.001
Age y, range	38–88	21–87	30–91	21–91	
Age y, median (IQR)	67 (56–73)	64 (55–74)	67 (58–79)	67 (57–76)	0.12^a^
Days of hospitalization					
Median (IQR)	3 (2–4)	4 (3–7)	9 (6–17)	6 (3–10)	< 0.001^a^
Disease day					
Median (IQR)	7 (3–10)	7 (6–10)	7 (5–10)	7 (5–10)	0.21^a^
Hospital re-admission—*n* (%)	1 (3)	6 (6)	8 (9)	15 (7)	0.15
Smoking—*n* (%)					
Active	0 (0)	5 (8)	1 (2)	6 (4)	
Former	9 (39)	27 (40)	23 (42)	59 (41)	
Never	5 (22)	13 (19)	14 (26)	32 (22)	
Not active, unknown past	9 (39)	22 (33)	17 (31)	48 (33)	0.71
Weight (kg), median (IQR)	82 (68–94)	86 (72–95)	86 (79–91)	85 (73–93)	0.12^a^
Medical history					
Comorbidity present—*n* (%)	31 (82)	90 (86)	72 (83)	193 (84)	0.98
Diabetes	7 (18)	24 (23)	27 (31)	58 (25)	0.10
Hypertension	11 (29)	38 (36)	37 (43)	86 (37)	0.14
Cardiovascular	10 (26)	28 (27)	35 (40)	73 (32)	0.06
Respirator	6 (16)	25 (24)	25 (29)	56 (24)	0.13
CNS	10 (26)	26 (25)	23 (26)	59 (26)	0.93
Renal	2 (5)	8 (8)	9 (10)	19 (8)	0.32
Malignancy	4 (11)	16 (15)	19 (22)	39 (17)	0.10
Autoimmune	2 (5)	11 (11)	14 (16)	27 (12)	0.07
Endocrine	4 (11)	5 (5)	7 (8)	16 (7)	0.88
Other	10 (26)	26 (25)	15 (17)	51 (22)	0.19

Disease day: days patient had COVID-19 symptoms until the day that the complete blood count was collected. Comorbidity present shows patients with at least one comorbidity. ‘Other’ comorbidities include gastrointestinal, musculoskeletal, urogenital, lipid disorders and Down syndrome. Smoking history *n* = 145. Weight *N* = 148. *p* values were calculated with a chi-square test for trend

*IQR* interquartile range

^a^ANOVA-F test for trend was used.

**Table 2:** Complete blood count of patients collected at day of admission or after positive COVID-19 test if patients were tested during hospital admission

	Mild	Moderate	Severe	*p* value univariate analysis^α^	*p* value multivariate analysis^β^
	(*n* = 38)	(*n* = 105)	(*n* = 87)		
Leukocytes × 10^9^ cells/L (median, IQR)	5.2 (4.0–7.5)	6.3 (4.7–8.0)	7.8 (5.7–10.8)	< 0.001	0.004
Immune granulocytes % (median, IQR)	0.01 (0.01–0.04)	0.02 (0.01–0.03)	0.03 (0.01–0.05)	0.019	0.063
Neutrophils × 10^9^ cells/L (median, IQR)	3.6 (2.3–5.5)	4.5 (3.1–6.4)	5.8 (4.0–8.1)	< 0.001	< 0.001
Lymphocytes × 10^9^ cells/L (median, IQR)	0.9 (0.7–1.5)	0.9 (0.7–1.3)	0.9 (0.6–1.2)	0.664	0.925
Monocytes × 10^9^ cells/L (median, IQR)	0.5 (0.4–0.7)	0.5 (0.3–0.7)	0.5 (0.3–0.7)	0.548	0.402
Eosinophils × 10^9^ cells/L (median, IQR)	0.01 (0.00–0.05)	0.00 (0.00–0.02)	0.00 (0.00–0.01)	0.004	0.028
Basophils × 10^9^ cells/L (median, IQR)	0.01 (0.01–0.02)	0.01 (0.01–0.02)	0.01 (0.01–0.02)	0.845	0.862
CRP mg/L (median, IQR)	26 (9–52)	77 (40–132)	140 (88–172)	< 0.001	< 0.001

Immune granulocytes *n* = 222

*IQR* interquartile range

^α^*p* values calculated with use of univariate linear regression model with blood count as dependent variable, log transformed in case of skewed distribution

^β^Multivariate linear regression model also taking into account the influence of age, sex and comorbidity

**Table 3:** Number of patients with lymphopenia and eosinopenia in the study population, stratified by COVID-19 severity group

	Mild	Moderate	Severe	Total	*p* value
	(*n* = 38)	(*n* = 105)	(*n* = 87)	(*n* = 230)	
Lymphopenia *n* (%)	12 (31.6)	31 (29.5)	33 (37.9)	76 (33.0)	0.34
Eosinopenia *n* (%)	28 (73.7)	91 (86.7)	82 (94.3)	201 (87.4)	0.002

Lymphopenia defined by lymphocyte counts below 0.8 × 10^9^ cells/L. Eosinopenia is defined by eosinophil counts below 0.04 × 10^9^ cells/L. *p* values calculated with *χ*^2^ test for trend.

## Discussion

In this study, we demonstrate that the majority (87%) of patients with COVID-19 presented with eosinopenia. The number of patients with eosinopenia rose with increasing severity of COVID-19. In the mild severity group, eosinopenia was present in 74% of the patients, whereas this was 87% in the moderate severity group and even 94% in the most severe group (*p* value 0.002). These numbers suggest a relation between eosinopenia and severity of COVID-19.

The role that eosinophils play in COVID-19 remains unclear. Eosinophils are leukocytes that normally account for a small percentage (1–3%) of peripheral blood leukocytes [5]. They have potentially pro-inflammatory effects due to their preformed granules, which are packed with cytotoxic proteins [5]. As eosinophils can become active during disease, the peripheral blood level can vary under different conditions. The cause of eosinopenia is presumably multifactorial and may be related to the migration of eosinophils into the peripheral tissues as seen in other viral infections. For example, in respiratory syncytial virus infections, an influx of eosinophils into the respiratory tract has been observed following acute infection [6]. Another explanation could be a decreased production of eosinophils in the bone marrow due to the inflammation or viral attach in the bone marrow, like it is the case in typhoid fever [7]. Further possible explanation could be the increased level of endogenous glucocorticoids which may influence peripheral eosinophil levels, therefore, patients with exogenous glucocorticoids use were excluded from this study [3, 8]. Furthermore, COVID-19 may causes CD8 T-cell depletion, which normally produces IL-5, amongst others [2]. IL-5 contributes to the proliferation and activation of eosinophils [9]. In addition, IL-13, which may induce eosinophilia in the lung depends largely on IL-5 [10]. If IL-5 production decreases, IL-13 could be less potent. IL-5 and IL-13 are coproduced by T helper 2 (T_H_2) cells. The role of T_H_2 cells in severe COVID-19 is however still unclear.

One of the limitations of this study is that we only know the number of disease days at the time of blood draw, not the amount of days between positive COVID-19 test and blood draw. The retrospective nature is another potential limitation of this study, however, the inclusion of 230 PCR confirmed COVID-19 patients and the novel data on the peripheral eosinophil levels in these patients are valuable in gaining more knowledge about COVID-19. Till date, only few studies focused on the role of eosinophils in COVID-19.

In conclusion, eosinopenia is present in the majority of patients with COVID-19 and appears to correlate with disease severity in our study. Further studies are required to learn what exactly the role of eosinophils are in COVID-19.

## Data Availability

Yes.

## Code availability

Yes.
